# Bis(5-amino-2-chloro­benzoato-κ*O*)triphenyl­anti­mony(V)

**DOI:** 10.1107/S1600536809043578

**Published:** 2009-10-28

**Authors:** Liyuan Wen, Handong Yin, Chuanhua Wang

**Affiliations:** a College of Chemistry and Chemical Engineering, Liaocheng University, Shandong 252059, People’s Republic of China

## Abstract

In the title compound, [Sb(C_6_H_5_)_3_(C_7_H_5_ClNO_2_)_2_], the Sb center has a distorted trigonal-bipyramidal geometry, with the O atoms of two carboxyl­ate groups in axial positions and the C atoms of the phenyl groups in equatorial positions. Intra­molecular C—H⋯O inter­actions occur. The mol­ecules are connected by inter­molecular N—H⋯O, N—H⋯N and C—H⋯O hydrogen-bonding inter­actions and C—H⋯π stacking inter­actions, forming a three-dimensional supra­molecular framework

## Related literature

For related structures, see: Yin *et al.* (2009 ▶); Wang *et al.* (2005 ▶).
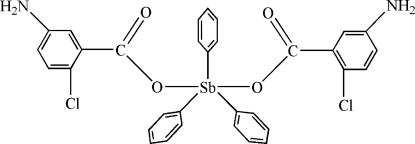


## Experimental

### 

#### Crystal data

[Sb(C_6_H_5_)_3_(C_7_H_5_ClNO_2_)_2_]
*M* *_r_* = 694.19
Monoclinic, 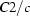

*a* = 30.683 (3) Å
*b* = 9.0128 (12) Å
*c* = 23.096 (3) Å
β = 106.161 (2)°
*V* = 6134.6 (12) Å^3^
*Z* = 8
Mo *K*α radiation
μ = 1.11 mm^−1^
*T* = 298 K
0.35 × 0.33 × 0.17 mm

#### Data collection

Siemens SMART CCD area-detector diffractometer
Absorption correction: multi-scan (*SADABS*; Sheldrick, 1996 ▶) *T*_min_ = 0.697, *T*_max_ = 0.833
15481 measured reflections
5409 independent reflections
3450 reflections with *I* > 2σ(*I*)
*R*_int_ = 0.046

#### Refinement

*R*[*F* ^2^ > 2σ(*F* ^2^)] = 0.038
*wR*(*F* ^2^) = 0.087
*S* = 1.04
5409 reflections
386 parameters
4 restraints
H atoms treated by a mixture of independent and constrained refinement
Δρ_max_ = 0.86 e Å^−3^
Δρ_min_ = −0.49 e Å^−3^

### 

Data collection: *SMART* (Siemens, 1996 ▶); cell refinement: *SAINT* (Siemens, 1996 ▶); data reduction: *SAINT*; program(s) used to solve structure: *SHELXS97* (Sheldrick, 2008 ▶); program(s) used to refine structure: *SHELXL97* (Sheldrick, 2008 ▶); molecular graphics: *SHELXTL* (Sheldrick, 2008 ▶); software used to prepare material for publication: *SHELXTL*.

## Supplementary Material

Crystal structure: contains datablocks I, global. DOI: 10.1107/S1600536809043578/gk2233sup1.cif

Structure factors: contains datablocks I. DOI: 10.1107/S1600536809043578/gk2233Isup2.hkl

Additional supplementary materials:  crystallographic information; 3D view; checkCIF report

## Figures and Tables

**Table:** 

Sb1—C21	2.098 (5)
Sb1—C15	2.108 (5)
Sb1—C27	2.109 (5)
Sb1—O3	2.125 (3)
Sb1—O1	2.137 (3)

**Table:** 

C21—Sb1—C15	109.59 (19)
C21—Sb1—C27	109.92 (19)
C15—Sb1—C27	140.49 (19)
C21—Sb1—O3	87.33 (16)
C15—Sb1—O3	92.36 (15)
C27—Sb1—O3	89.15 (15)
C21—Sb1—O1	87.45 (16)
C15—Sb1—O1	87.74 (15)
C27—Sb1—O1	94.27 (15)
O3—Sb1—O1	174.49 (12)

**Table 2:** Hydrogen-bond geometry (Å, °)

*D*—H⋯*A*	*D*—H	H⋯*A*	*D*⋯*A*	*D*—H⋯*A*
C32—H32⋯O2	0.93	2.39	3.105 (6)	134
C20—H20⋯O4	0.93	2.47	3.120 (6)	127
N1—H1*B*⋯N2^i^	0.865 (19)	2.43 (3)	3.211 (8)	150 (5)
N1—H1*A*⋯O2^ii^	0.86 (2)	2.43 (5)	3.114 (6)	137 (5)
N2—H2*A*⋯N1^iii^	0.847 (19)	2.44 (2)	3.272 (7)	169 (5)
N2—H2*B*⋯O4^iv^	0.847 (19)	2.36 (3)	3.151 (6)	156 (5)
C14—H14⋯O4^iv^	0.93	2.58	3.369 (6)	143
C12—H12⋯O2^v^	0.93	2.56	3.433 (6)	157
C23—H23⋯*Cg*1^vi^	0.93	2.72	3.567 (7)	151

Symmetry codes: (i) 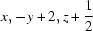; (ii) 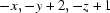; (iii) 
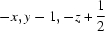
; (iv) 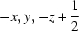; (v) 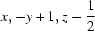; (vi) 
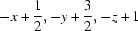
. *Cg*1 is the centroid of the C27–C32 ring.

**Fig. 1.**
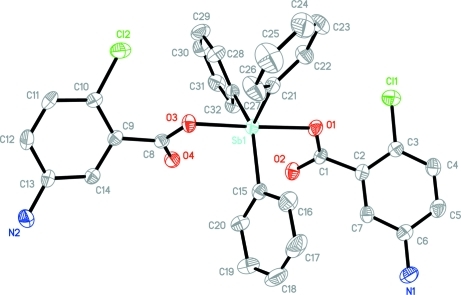
The molecular structure of the compound, showing 50% probability displacement ellipsoids. H atoms have been omitted for clarity.

**Fig. 2.**
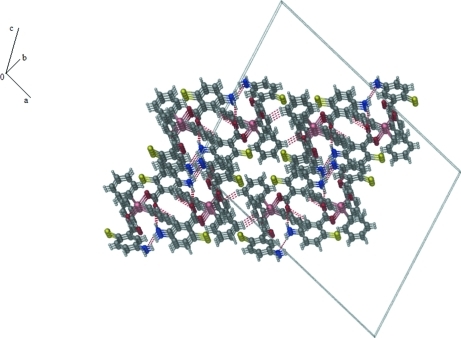
View of the three-dimensional supramolecular network structure in the title compound.

## supplementary crystallographic information

### Comment

Recently, the chemistry of antimony complexes derived from carboxylates has become an active area of research due to the biological perspective and their versatile bonding modes, the striking structural possibilities ranging from discrete monomeric structures to supramolecular assemblies (Yin *et al.* 2009). As a part of our ongoing investigations in ths field we have synthesized the title compound and determined its crystal structure. As is shown in Fig. 1, the central antimony atom is five-coordinated with a slightly distorted trigonal bipyramidal geometry. Around the central Sb1 atom, C15, C21 and C27 occupy the equatorial plane, while O1 and O3 lie in axial sites. The Sb—O bond distances (Sb1—O1 = 2.137 (3) Å; Sb1—O3 = 2.125 (3) Å) (Table 1) are comparable to those found in organoantimony arylhydroxmates (Wang *et al.* 2005). The Sb—C bond distances (Sb1—C15 = 2.108 (5) Å; Sb1—C21 = 2.098 (5) Å; Sb1—C27 = 2.109 (5) Å) of the compound lie within the normal range for Sb—C (phenyl) bonds (2.10–2.13 Å). The supramolecular structure of the title compound results from intermolecular C—H···O, N—H···O and N—H···N hydrogen-bonding interactions and C—H···Π stacking interactions (Fig. 2, Table 2) assembling the molecules into a three-dimensional supramolecular frameworks

### Experimental

The reaction was carried out under nitrogen atmosphere. 5-Amino-2-chlorobenzoic acid (1 mmol) and sodium ethoxide (1.2 mmol) were added to a stirred solution of methanol (30 ml) in a Schlenk flask and stirred for 0.5 h. Triphenylantimony dichloride (0.5 mmol) was then added to the reactor and the reaction mixture was stirred for 12 h at room temperature. The resulting clear solution was evaporated under vacuum. The product was crystallized from a mixture of ether/n-hexane (1:1) to yield colourless blocks of the title compound (yield 80%). Anal. Calcd (%) for C_32_H_25_Cl_2_N_2_O_4_Sb (Mr = 694.19): C, 55.37; H, 3.63; Cl, 10.21; N, 4.04. Found (%): C, 55.31; H, 3.76; Cl, 10.31; N, 4.15.

### Refinement

The N-bound H atoms were located in a difference Fourier map. In the refinement process the N-H bond lengths were restrained to 0.85 (2) Å and isotropic displacement parameters of these H atoms  were freely refined. Other H atoms were positioned geometrically, with C—H = 0.93 Å and refined with a riding model; *U*_iso_(H) = 1.2 *U*_eq_ (C).

### Crystal data

**Table:**

[Sb(C_6_H_5_)_3_(C_7_H_5_ClNO_2_)_2_]	*F*(000) = 2784
*M**_r_* = 694.19	*D*_x_ = 1.503 Mg m^−^^3^
Monoclinic, *C*2/*c*	Mo *K*α radiation, λ = 0.71073 Å
Hall symbol: -C 2yc	Cell parameters from 3588 reflections
*a* = 30.683 (3) Å	θ = 2.4–23.7°
*b* = 9.0128 (12) Å	µ = 1.11 mm^−^^1^
*c* = 23.096 (3) Å	*T* = 298 K
β = 106.161 (2)°	Block, colourless
*V* = 6134.6 (12) Å^3^	0.35 × 0.33 × 0.17 mm
*Z* = 8	

### Data collection

**Table:**

Siemens SMART CCD area-detector diffractometer	5409 independent reflections
Radiation source: fine-focus sealed tube	3450 reflections with *I* > 2σ(*I*)
graphite	*R*_int_ = 0.046
φ and ω scans	θ_max_ = 25.0°, θ_min_ = 1.4°
Absorption correction: multi-scan (*SADABS*; Sheldrick, 1996)	*h* = −25→36
*T*_min_ = 0.697, *T*_max_ = 0.833	*k* = −10→10
15481 measured reflections	*l* = −27→22

### Refinement

**Table:**

Refinement on *F*^2^	Primary atom site location: structure-invariant direct methods
Least-squares matrix: full	Secondary atom site location: difference Fourier map
*R*[*F*^2^ > 2σ(*F*^2^)] = 0.038	Hydrogen site location: inferred from neighbouring sites
*wR*(*F*^2^) = 0.087	H atoms treated by a mixture of independent and constrained refinement
*S* = 1.04	*w* = 1/[σ^2^(*F*_o_^2^) + (0.0252*P*)^2^ + 12.9995*P*] where *P* = (*F*_o_^2^ + 2*F*_c_^2^)/3
5409 reflections	(Δ/σ)_max_ < 0.001
386 parameters	Δρ_max_ = 0.86 e Å^−^^3^
4 restraints	Δρ_min_ = −0.49 e Å^−^^3^

### Fractional atomic coordinates and isotropic or equivalent isotropic displacement parameters (Å^2^)

**Table:**

	*x*	*y*	*z*	*U*_iso_*/*U*_eq_	
Sb1	0.133450 (10)	0.75719 (4)	0.387976 (14)	0.03529 (11)	
Cl1	0.19341 (5)	0.92982 (17)	0.61118 (7)	0.0683 (4)	
Cl2	0.17565 (5)	0.4095 (2)	0.25676 (8)	0.0998 (7)	
N1	0.02031 (18)	1.2629 (6)	0.5343 (2)	0.0590 (12)	
N2	−0.00974 (17)	0.4432 (6)	0.0918 (2)	0.0567 (13)	
O1	0.14690 (11)	0.8704 (3)	0.47239 (14)	0.0427 (8)	
O2	0.08342 (11)	0.7652 (4)	0.48081 (14)	0.0478 (8)	
O3	0.12400 (10)	0.6602 (3)	0.30150 (13)	0.0411 (8)	
O4	0.06817 (10)	0.5306 (4)	0.32076 (15)	0.0445 (9)	
C1	0.11308 (17)	0.8607 (5)	0.4962 (2)	0.0390 (12)	
C2	0.10837 (16)	0.9804 (5)	0.5384 (2)	0.0382 (12)	
C3	0.14220 (16)	1.0253 (5)	0.5887 (2)	0.0432 (13)	
C4	0.13472 (19)	1.1428 (6)	0.6237 (2)	0.0531 (14)	
H4	0.1570	1.1696	0.6585	0.064*	
C5	0.09432 (19)	1.2197 (6)	0.6068 (2)	0.0571 (15)	
H5	0.0898	1.2997	0.6299	0.069*	
C6	0.06032 (18)	1.1792 (6)	0.5558 (2)	0.0469 (13)	
C7	0.06747 (17)	1.0588 (5)	0.5228 (2)	0.0426 (12)	
H7	0.0445	1.0290	0.4893	0.051*	
C8	0.09252 (16)	0.5591 (5)	0.2885 (2)	0.0360 (11)	
C9	0.08637 (16)	0.4847 (5)	0.2287 (2)	0.0367 (11)	
C10	0.12097 (16)	0.4236 (6)	0.2091 (2)	0.0524 (14)	
C11	0.11202 (19)	0.3647 (6)	0.1518 (3)	0.0648 (17)	
H11	0.1353	0.3213	0.1392	0.078*	
C12	0.06878 (19)	0.3698 (6)	0.1131 (2)	0.0560 (15)	
H12	0.0635	0.3331	0.0741	0.067*	
C13	0.03327 (16)	0.4285 (5)	0.1315 (2)	0.0388 (12)	
C14	0.04275 (16)	0.4845 (5)	0.1899 (2)	0.0391 (12)	
H14	0.0191	0.5231	0.2032	0.047*	
C15	0.07540 (17)	0.8918 (5)	0.3575 (2)	0.0408 (12)	
C16	0.0831 (2)	1.0412 (6)	0.3515 (3)	0.0618 (16)	
H16	0.1126	1.0768	0.3595	0.074*	
C17	0.0468 (3)	1.1380 (7)	0.3337 (3)	0.084 (2)	
H17	0.0518	1.2384	0.3286	0.100*	
C18	0.0039 (3)	1.0854 (8)	0.3236 (3)	0.080 (2)	
H18	−0.0203	1.1514	0.3129	0.095*	
C19	−0.00454 (19)	0.9387 (7)	0.3289 (3)	0.0665 (17)	
H19	−0.0342	0.9047	0.3212	0.080*	
C20	0.03162 (18)	0.8400 (6)	0.3460 (2)	0.0526 (14)	
H20	0.0262	0.7394	0.3497	0.063*	
C21	0.18995 (16)	0.8670 (5)	0.3742 (2)	0.0413 (12)	
C22	0.22768 (18)	0.8915 (6)	0.4213 (3)	0.0553 (15)	
H22	0.2283	0.8595	0.4598	0.066*	
C23	0.26495 (19)	0.9639 (7)	0.4118 (3)	0.0706 (18)	
H23	0.2908	0.9784	0.4437	0.085*	
C24	0.2638 (2)	1.0132 (8)	0.3563 (4)	0.085 (2)	
H24	0.2887	1.0628	0.3503	0.102*	
C25	0.2264 (2)	0.9912 (8)	0.3089 (3)	0.098 (3)	
H25	0.2259	1.0255	0.2708	0.117*	
C26	0.18914 (19)	0.9175 (7)	0.3177 (3)	0.0725 (19)	
H26	0.1636	0.9022	0.2854	0.087*	
C27	0.15298 (16)	0.5477 (5)	0.4274 (2)	0.0375 (12)	
C28	0.18933 (18)	0.4839 (6)	0.4126 (2)	0.0545 (15)	
H28	0.2048	0.5363	0.3898	0.065*	
C29	0.2027 (2)	0.3410 (7)	0.4322 (3)	0.0680 (17)	
H29	0.2274	0.2980	0.4228	0.082*	
C30	0.1797 (2)	0.2636 (6)	0.4651 (3)	0.0632 (16)	
H30	0.1886	0.1676	0.4779	0.076*	
C31	0.14364 (19)	0.3273 (6)	0.4793 (3)	0.0584 (16)	
H31	0.1280	0.2738	0.5015	0.070*	
C32	0.12994 (16)	0.4711 (5)	0.4609 (2)	0.0447 (13)	
H32	0.1056	0.5144	0.4713	0.054*	
H1A	−0.0042 (13)	1.213 (6)	0.521 (3)	0.09 (3)*	
H2A	−0.0141 (17)	0.386 (5)	0.0615 (16)	0.061 (19)*	
H1B	0.0182 (19)	1.324 (5)	0.5623 (19)	0.08 (2)*	
H2B	−0.0315 (12)	0.450 (6)	0.108 (2)	0.057 (19)*	

### Atomic displacement parameters (Å^2^)

**Table:**

	*U*^11^	*U*^22^	*U*^33^	*U*^12^	*U*^13^	*U*^23^
Sb1	0.03935 (18)	0.03370 (18)	0.03511 (18)	−0.00248 (17)	0.01417 (13)	−0.00089 (18)
Cl1	0.0535 (9)	0.0761 (11)	0.0696 (11)	0.0103 (8)	0.0079 (7)	−0.0040 (8)
Cl2	0.0513 (10)	0.1624 (19)	0.0777 (13)	0.0366 (11)	0.0047 (8)	−0.0392 (12)
N1	0.059 (3)	0.057 (3)	0.064 (3)	0.010 (3)	0.023 (3)	0.000 (3)
N2	0.049 (3)	0.077 (4)	0.043 (3)	−0.002 (3)	0.010 (3)	−0.014 (3)
O1	0.047 (2)	0.047 (2)	0.038 (2)	0.0001 (16)	0.0193 (17)	−0.0075 (16)
O2	0.058 (2)	0.039 (2)	0.050 (2)	−0.0073 (18)	0.0201 (17)	−0.0049 (18)
O3	0.049 (2)	0.0379 (19)	0.038 (2)	−0.0100 (16)	0.0161 (16)	−0.0050 (16)
O4	0.042 (2)	0.055 (2)	0.040 (2)	−0.0035 (17)	0.0166 (17)	−0.0031 (17)
C1	0.050 (3)	0.031 (3)	0.038 (3)	0.003 (2)	0.014 (3)	0.001 (2)
C2	0.049 (3)	0.035 (3)	0.036 (3)	−0.005 (2)	0.021 (2)	0.001 (2)
C3	0.047 (3)	0.045 (3)	0.039 (3)	0.002 (2)	0.014 (3)	0.003 (2)
C4	0.063 (4)	0.055 (4)	0.041 (3)	−0.003 (3)	0.014 (3)	−0.007 (3)
C5	0.066 (4)	0.061 (4)	0.050 (4)	0.000 (3)	0.025 (3)	−0.013 (3)
C6	0.050 (3)	0.042 (3)	0.054 (4)	0.000 (3)	0.023 (3)	0.004 (3)
C7	0.054 (3)	0.040 (3)	0.034 (3)	−0.005 (2)	0.012 (2)	−0.001 (2)
C8	0.038 (3)	0.033 (3)	0.038 (3)	0.004 (2)	0.012 (2)	0.003 (2)
C9	0.039 (3)	0.036 (3)	0.039 (3)	0.000 (2)	0.018 (2)	0.001 (2)
C10	0.041 (3)	0.066 (4)	0.052 (4)	0.009 (3)	0.014 (3)	−0.012 (3)
C11	0.060 (4)	0.077 (4)	0.064 (4)	0.015 (3)	0.027 (3)	−0.027 (3)
C12	0.064 (4)	0.059 (4)	0.045 (4)	−0.003 (3)	0.016 (3)	−0.021 (3)
C13	0.046 (3)	0.037 (3)	0.037 (3)	−0.005 (2)	0.017 (3)	−0.006 (2)
C14	0.041 (3)	0.036 (3)	0.045 (3)	−0.002 (2)	0.019 (2)	−0.001 (2)
C15	0.050 (3)	0.040 (3)	0.034 (3)	0.002 (2)	0.014 (2)	0.000 (2)
C16	0.072 (4)	0.038 (3)	0.073 (5)	0.002 (3)	0.018 (3)	0.006 (3)
C17	0.118 (6)	0.037 (4)	0.092 (6)	0.025 (4)	0.024 (5)	0.005 (3)
C18	0.092 (5)	0.072 (5)	0.074 (5)	0.044 (4)	0.023 (4)	0.006 (4)
C19	0.056 (4)	0.070 (4)	0.077 (5)	0.019 (3)	0.023 (3)	0.008 (4)
C20	0.052 (4)	0.046 (3)	0.061 (4)	0.011 (3)	0.018 (3)	0.005 (3)
C21	0.045 (3)	0.037 (3)	0.047 (3)	−0.006 (2)	0.021 (3)	−0.006 (2)
C22	0.052 (4)	0.061 (4)	0.053 (4)	−0.010 (3)	0.013 (3)	−0.004 (3)
C23	0.045 (4)	0.085 (5)	0.078 (5)	−0.019 (3)	0.010 (3)	−0.020 (4)
C24	0.073 (5)	0.090 (5)	0.105 (6)	−0.037 (4)	0.044 (5)	−0.015 (5)
C25	0.099 (6)	0.126 (7)	0.080 (6)	−0.046 (5)	0.043 (5)	0.012 (5)
C26	0.063 (4)	0.101 (5)	0.057 (4)	−0.040 (4)	0.022 (3)	0.006 (4)
C27	0.042 (3)	0.033 (3)	0.034 (3)	0.002 (2)	0.004 (2)	0.001 (2)
C28	0.060 (4)	0.050 (3)	0.060 (4)	0.012 (3)	0.027 (3)	0.005 (3)
C29	0.066 (4)	0.064 (4)	0.073 (5)	0.027 (3)	0.018 (3)	0.003 (4)
C30	0.076 (4)	0.037 (3)	0.068 (4)	0.007 (3)	0.005 (3)	0.008 (3)
C31	0.057 (4)	0.044 (3)	0.070 (4)	−0.009 (3)	0.010 (3)	0.011 (3)
C32	0.044 (3)	0.041 (3)	0.049 (3)	−0.003 (2)	0.011 (3)	0.006 (3)

### Geometric parameters (Å, °)

**Table:**

Sb1—C21	2.098 (5)	C13—C14	1.392 (6)
Sb1—C15	2.108 (5)	C14—H14	0.9300
Sb1—C27	2.109 (5)	C15—C20	1.376 (6)
Sb1—O3	2.125 (3)	C15—C16	1.381 (6)
Sb1—O1	2.137 (3)	C16—C17	1.383 (7)
Cl1—C3	1.739 (5)	C16—H16	0.9300
Cl2—C10	1.736 (5)	C17—C18	1.357 (8)
N1—C6	1.408 (7)	C17—H17	0.9300
N1—H1A	0.86 (2)	C18—C19	1.360 (8)
N1—H1B	0.865 (19)	C18—H18	0.9300
N2—C13	1.388 (6)	C19—C20	1.391 (7)
N2—H2A	0.847 (19)	C19—H19	0.9300
N2—H2B	0.847 (19)	C20—H20	0.9300
O1—C1	1.305 (5)	C21—C22	1.369 (6)
O2—C1	1.231 (5)	C21—C26	1.375 (7)
O3—C8	1.301 (5)	C22—C23	1.386 (7)
O4—C8	1.220 (5)	C22—H22	0.9300
C1—C2	1.488 (6)	C23—C24	1.348 (8)
C2—C3	1.385 (6)	C23—H23	0.9300
C2—C7	1.397 (6)	C24—C25	1.361 (9)
C3—C4	1.390 (7)	C24—H24	0.9300
C4—C5	1.378 (7)	C25—C26	1.385 (7)
C4—H4	0.9300	C25—H25	0.9300
C5—C6	1.387 (7)	C26—H26	0.9300
C5—H5	0.9300	C27—C32	1.372 (6)
C6—C7	1.378 (6)	C27—C28	1.379 (6)
C7—H7	0.9300	C28—C29	1.388 (7)
C8—C9	1.499 (6)	C28—H28	0.9300
C9—C10	1.379 (6)	C29—C30	1.364 (8)
C9—C14	1.388 (6)	C29—H29	0.9300
C10—C11	1.381 (7)	C30—C31	1.366 (7)
C11—C12	1.378 (7)	C30—H30	0.9300
C11—H11	0.9300	C31—C32	1.391 (7)
C12—C13	1.380 (6)	C31—H31	0.9300
C12—H12	0.9300	C32—H32	0.9300
			
C21—Sb1—C15	109.59 (19)	C9—C14—C13	122.0 (5)
C21—Sb1—C27	109.92 (19)	C9—C14—H14	119.0
C15—Sb1—C27	140.49 (19)	C13—C14—H14	119.0
C21—Sb1—O3	87.33 (16)	C20—C15—C16	119.7 (5)
C15—Sb1—O3	92.36 (15)	C20—C15—Sb1	123.9 (4)
C27—Sb1—O3	89.15 (15)	C16—C15—Sb1	116.3 (4)
C21—Sb1—O1	87.45 (16)	C15—C16—C17	120.0 (6)
C15—Sb1—O1	87.74 (15)	C15—C16—H16	120.0
C27—Sb1—O1	94.27 (15)	C17—C16—H16	120.0
O3—Sb1—O1	174.49 (12)	C18—C17—C16	119.5 (6)
C6—N1—H1A	116 (4)	C18—C17—H17	120.3
C6—N1—H1B	108 (4)	C16—C17—H17	120.3
H1A—N1—H1B	112 (6)	C17—C18—C19	121.6 (6)
C13—N2—H2A	113 (4)	C17—C18—H18	119.2
C13—N2—H2B	116 (4)	C19—C18—H18	119.2
H2A—N2—H2B	116 (5)	C18—C19—C20	119.4 (6)
C1—O1—Sb1	112.2 (3)	C18—C19—H19	120.3
C8—O3—Sb1	113.8 (3)	C20—C19—H19	120.3
O2—C1—O1	122.4 (4)	C15—C20—C19	119.8 (5)
O2—C1—C2	119.8 (5)	C15—C20—H20	120.1
O1—C1—C2	117.5 (4)	C19—C20—H20	120.1
C3—C2—C7	118.5 (4)	C22—C21—C26	119.3 (5)
C3—C2—C1	125.1 (4)	C22—C21—Sb1	120.5 (4)
C7—C2—C1	116.2 (4)	C26—C21—Sb1	120.2 (4)
C2—C3—C4	120.3 (5)	C21—C22—C23	120.1 (5)
C2—C3—Cl1	120.6 (4)	C21—C22—H22	119.9
C4—C3—Cl1	119.0 (4)	C23—C22—H22	119.9
C5—C4—C3	119.9 (5)	C24—C23—C22	120.1 (6)
C5—C4—H4	120.0	C24—C23—H23	120.0
C3—C4—H4	120.0	C22—C23—H23	120.0
C4—C5—C6	120.9 (5)	C23—C24—C25	120.6 (6)
C4—C5—H5	119.6	C23—C24—H24	119.7
C6—C5—H5	119.6	C25—C24—H24	119.7
C7—C6—C5	118.6 (5)	C24—C25—C26	119.8 (7)
C7—C6—N1	118.9 (5)	C24—C25—H25	120.1
C5—C6—N1	122.3 (5)	C26—C25—H25	120.1
C6—C7—C2	121.7 (5)	C21—C26—C25	120.0 (6)
C6—C7—H7	119.1	C21—C26—H26	120.0
C2—C7—H7	119.1	C25—C26—H26	120.0
O4—C8—O3	123.2 (5)	C32—C27—C28	120.6 (5)
O4—C8—C9	121.8 (4)	C32—C27—Sb1	124.4 (4)
O3—C8—C9	114.9 (4)	C28—C27—Sb1	114.8 (4)
C10—C9—C14	118.6 (5)	C27—C28—C29	119.5 (5)
C10—C9—C8	124.7 (4)	C27—C28—H28	120.2
C14—C9—C8	116.6 (4)	C29—C28—H28	120.2
C9—C10—C11	120.1 (5)	C30—C29—C28	120.3 (6)
C9—C10—Cl2	121.1 (4)	C30—C29—H29	119.9
C11—C10—Cl2	118.6 (4)	C28—C29—H29	119.9
C12—C11—C10	120.5 (5)	C29—C30—C31	119.8 (5)
C12—C11—H11	119.8	C29—C30—H30	120.1
C10—C11—H11	119.8	C31—C30—H30	120.1
C11—C12—C13	120.9 (5)	C30—C31—C32	121.0 (6)
C11—C12—H12	119.6	C30—C31—H31	119.5
C13—C12—H12	119.6	C32—C31—H31	119.5
C12—C13—N2	121.3 (5)	C27—C32—C31	118.7 (5)
C12—C13—C14	117.8 (5)	C27—C32—H32	120.6
N2—C13—C14	120.6 (5)	C31—C32—H32	120.6

### Hydrogen-bond geometry (Å, °)

**Table:**

*D*—H···*A*	*D*—H	H···*A*	*D*···*A*	*D*—H···*A*
C32—H32···O2	0.93	2.39	3.105 (6)	134
C20—H20···O4	0.93	2.47	3.120 (6)	127
N1—H1B···N2^i^	0.87 (2)	2.43 (3)	3.211 (8)	150 (5)
N1—H1A···O2^ii^	0.86 (2)	2.43 (5)	3.114 (6)	137 (5)
N2—H2A···N1^iii^	0.85 (2)	2.44 (2)	3.272 (7)	169 (5)
N2—H2B···O4^iv^	0.85 (2)	2.36 (3)	3.151 (6)	156 (5)
C14—H14···O4^iv^	0.93	2.58	3.369 (6)	143
C12—H12···O2^v^	0.93	2.56	3.433 (6)	157
C23—H23···Cg1^vi^	0.93	2.72	3.567 (7)	151

Symmetry codes: (i) *x*, −*y*+2, *z*+1/2; (ii) −*x*, −*y*+2, −*z*+1; (iii) −*x*, *y*−1, −*z*+1/2; (iv) −*x*, *y*, −*z*+1/2; (v) *x*, −*y*+1, *z*−1/2; (vi) −*x*+1/2, −*y*+3/2, −*z*+1.
